# New Orf Virus (Parapoxvirus) Recombinant Expressing H5 Hemagglutinin Protects Mice against H5N1 and H1N1 Influenza A Virus

**DOI:** 10.1371/journal.pone.0083802

**Published:** 2013-12-20

**Authors:** Jörg Rohde, Ralf Amann, Hanns-Joachim Rziha

**Affiliations:** Institute of Immunology, Friedrich-Loeffler-Institute, Federal Research Institute for Animal Health, Island of Riems, Greifswald, Germany; Thomas Jefferson University, United States of America

## Abstract

Previously we demonstrated the versatile utility of the *Parapoxvirus* Orf virus (ORFV) as a vector platform for the development of potent recombinant vaccines. In this study we present the generation of new ORFV recombinants expressing the hemagglutinin (HA) or nucleoprotein (NP) of the highly pathogenic avian influenza virus (HPAIV) H5N1. Correct foreign gene expression was examined *in vitro* by immunofluorescence, Western blotting and flow cytometry. The protective potential of both recombinants was evaluated in the mouse challenge model. Despite adequate expression of NP, the recombinant D1701-V-NPh5 completely failed to protect mice from lethal challenge. However, the H5 HA-expressing recombinant D1701-V-HAh5n mediated solid protection in a dose-dependent manner. Two intramuscular (i.m.) injections of the HA-expressing recombinant protected all animals from lethal HPAIV infection without loss of body weight. Notably, the immunized mice resisted cross-clade H5N1 and heterologous H1N1 (strain PR8) influenza virus challenge. *In vivo* antibody-mediated depletion of CD4-positive and/or CD8-posititve T-cell subpopulations during immunization and/or challenge infection implicated the relevance of CD4-positive T-cells for induction of protective immunity by D1701-V-HAh5n, whereas the absence of CD8-positive T-cells did not significantly influence protection. In summary, this study validates the potential of the ORFV vectored vaccines also to combat HPAIV.

## Introduction

Influenza A virus is a member of the *Orthomyxoviridae* and can infect numerous hosts, including aquatic birds, poultry, swine and humans (for review [1]). Its negative-sense, single-stranded RNA genome is composed of eight gene segments encoding the viral proteins. The genetic variation of the surface glycoproteins hemagglutinin (HA) and neuraminidase (NA) is the basis for further subtyping influenza A viruses in H1 to H16 and N1–N9, respectively [2], and a novel subtype H17N10 has recently been detected in bats [3], [4]. Cross-species transmission of influenza viruses to humans has been documented frequently, and in 2009 the new H1N1 influenza A virus (pH1N1) resulted from recombination of gene segments from human, swine and avian influenza A virus causing a new pandemic human flu [5]. The highly pathogenic avian influenza virus (HPAIV) H5N1 has caused outbreaks in wild birds and poultry leading to severe, fatal disease [6], and transmission from birds to humans was reported [1], [7]. The World Health Organization registers approximately 600 confirmed human H5N1 virus infections, approximately 60% resulting in death (WHO, August 2013; http://www.who.int/influenza/human_animal_interface/EN_GIP_20130829CumulativeNumberH5N1cases.pdf). Thus, serious concerns exist about the emergence of a pandemic H5N1 strain transmissible between humans. The trimeric HA is an important viral factor determining virulence, host tropism and transmission of influenza A virus [8], [9], [10], [11]. For entering the host cell the HA0 precursor form of the trimeric HA must be proteolytically cleaved into HA1, which binds to sialic acid-containing host cell receptors, and into HA2, which mediates membrane fusion. This cleavage site differs amongst HA subtypes, which in part, can determine the degree of virulence (for review [12]). Influenza virus infections can be effectively controlled and prevented by vaccination. Currently, inactivated vaccines are produced according to the HA and NA subtypes of circulating virus strains. Virus-neutralizing and receptor-blocking antibodies directed against HA1, the globular head of HA, can mediate sterilizing immunity provided that they have the proper strain-specificity. However, the rapid mutation rate of NA and of HA1 can impede the production of effective vaccines matching currently circulating virus types. Therefore, several attempts are reported for the generation of effective, more universal influenza virus vaccines (reviewed in [13]). Plasmid DNA vaccines expressing consensus sequences of HA and NA mounted cross-reactive cellular and humoral immune responses [14], [15] and were able to protect mice against divergent H5N1 strains [16]. Other approaches comprise the development of headless constructs, also to limit the suggested immunodominance of the globular head of HA [17]. Recent reports on the construction of various chimeric head and stalk HA proteins or functional influenza viruses expressing those chimeras offer another strategy for cross-protecting vaccines [18], [19].

Besides the humoral immune response against Influenza virus, T-cells that either eliminate infected cells or help B-cells to mount a more rapid and efficient neutralizing antibody response are also important to relieve the disease [20]. Especially cytolytic and cytokine-secreting T-cells directed to conserved influenza virus proteins, like the nucleoprotein (NP) or matrix protein (M1), can represent effectors in protective immunity [21], [22], [23] and are considered another promising approach for the development of more universal influenza vaccines [24], [25], [26]. HA epitopes, which are recognized by virus-specific human and mouse CD8-positive cytolytic T-cells, have also been identified (reviewed in [27]). The role of additional viral targets in adaptive, protective immunity against influenza A virus has recently been reviewed comprehensively [28], [29].

Various strategies are pursued to develop improved, safe, effective and cross-protecting vaccines not only against H5N1 strains but also against different influenza A virus subtypes. Those approaches comprise the generation of baculovirus-based multivalent vaccines [30] or self-assembling viral-like particles [31], [32], or DNA vaccines preferentially now in prime boost combinations with e.g. adenovirus recombinants [13]. The efficient and fast technology of reverse genetics allows the safe and effective creation of recombinant or attenuated influenza viruses with almost every desired gene alteration and constellation (reviewed in [33]). Moreover, attenuated influenza virus designed by a synthetic engineering approach to recode and synthesize the viral genome induced protective immunity in mice [34]. Finally, very recently the successful vaccination with optimized mRNA of HA, NA, and NP was reported, which stimulated T- and B-cell dependent protection against influenza A H1N1, H3N2 and H5N1 viruses [35]. Poxvirus-vectored vaccines are attractive due to the possibility for inserting multiple antigens by established methods, and their potential of rapid stimulation of good humoral and cell-mediated immune responses also mediating protection against e.g. HPAIV challenge infection [36], [37], [38], [39]. For safety reasons attenuated or replication-deficient poxviral vectors have been developed and used to mount protective immune responses against different influenza A virus subtypes [40], [41].

The *Orf Virus* (ORFV) from the genus *Parapoxviridae* (*PPV*) represents a promising candidate for novel vectored vaccines [42], [43], [44], [45], [46], [47], [48], [49]. ORFV has a very restricted host range *in vivo and in vitro*, a restricted skin tropism and an absence of systemic infection [50]. Ideal vector vaccine properties are the short-lived ORFV vector-specific immunity allowing repeated immunizations, and still not entirely understood immunomodulating properties, which lead to the induction of strong innate and adaptive Th1-Th2 balanced immune responses [44], [45], [50], [51]. The inserted foreign genes are regulated by an early ORFV promoter, which results in the induction of foreign antigen-specific immunity without the need of replication and multiplication of mature, infectious ORFV.

The present study describes the generation of new ORFV recombinants expressing the HPAIV genes H5 HA (D1701-V-HAh5n) or H5 NP (D1701-V-NPh5). After demonstrating proper expression of the inserted HPAIV genes, the protective potential of both recombinants was investigated by challenge infection of mice. Whereas the HA-expressing recombinant was able to protect all mice against lethal H5N1 virus challenge, the NP-expressing recombinant failed to mount protective immunity. Intramuscular (i.m.) immunization with D1701-V-HAh5n mediated cross-clade (H5N1 clades 1, 2.2.2, and 2.2.3) and heterosubtypic (H1N1) protection in different mouse strains. *In vivo* T-cell depletion experiments and a dose dependent increase of H5 HA-specific antibodies indicated that both arms of the immune response seem to be essential for protection after immunization with the HA-expressing ORFV recombinant.

## Materials and Methods

### Ethics statement

All animal studies were reviewed and approved by the local authorities (Regional council of Tuebingen) and were carried out in strict accordance with the regulations of the German animal welfare law set forth by this authority (permit number FLI 250/10).

### Cells and viruses

Propagation and titration of ORFV in Vero cells has been described earlier [44]. The highly pathogenic H5N1 avian influenza A viruses (HPAIV) A/Mallard/Bavaria/1/2006 (MB1, clade 2.2.1), A/mute swan/Germany/R1349/07 (SN1, clade 2.2.3), and the H1N1 human influenza A virus A/Puerto Rico/8/34 (PR8) were kindly provided by O. Planz (Univ. Tübingen, Dep. Immunology) and L. Stitz (Friedrich-Loeffler-Institut, Germany). The HPAIV were propagated and titrated as described [20]. For inactivation, the MB1 virus was incubated with 0.02% formalin at 4°C for three days and then stored at −20°C.

### Generation and selection of new ORFV recombinants

The HA coding sequence of H5N1 influenza A strain Vietnam/1203/2004 (Acc. no. AY818135) and the NP coding sequence of strain MB1 (Acc. no. DQ792924) were chemically synthesized by GeneArt (Regensburg, Germany) changing poxviral early transcript stop motifs (TTTTTCT) by silent mutations from codon TTT to TTC. In addition, new restriction sites were added to the 5′ and 3′ ends of both genes allowing to clone the HA gene as a HindIII – BamHI fragment and the NP gene as a KpnI – EcoRI fragment into plasmid pdV-Rec1 [44]. Correct insertion of the AIV genes into the obtained transfer plasmids pdV-HAh5n3 and pdV-NPh5n were tested by DNA-sequencing and restriction enzyme analysis (data not shown). Electroporation of *LacZ* positive ORFV D1701-VrV-infected Vero cells (moi 0.1–0.2) with 2 µg pdV-HAh5n3 DNA or pdV-NPh5n DNA, respectively, and selection of the new ORFV recombinants was described recently [46]. Single plaque PCR was used to screen virus progeny positive for the HA or the NP gene and negative for the *LacZ* gene of the parental virus D1701-VrV. Oligonucleotides used as PCR primers were purchased from Metabion (Martinsried, Germany). H5 HA-specific amplification (459 bp) was achieved with 3.8 pmol primer HA5Fn 5′-GTG AGC AGC GCA TGT CCT TAC CAG-3′ and 3.8 pmol primer HA5-Rnn 5′-CTC CCA TAG GGG TCT GGC ACT TTG-3′, NP-specific amplification (452 bp) with 4 pmol primer NP5-F 5′-GGA GGA TTT GGC GTC AAG CGA AC-3′ and 3.8 pmol primer NP5-R 5′-CTC TCA GGA TGA GTG CAG ACC TTG-3′. The PCR reactions contained 2X Reddy mix (ABgene, Fisher Scientific, Germany) and were denatured at 98°C for 2 minutes followed by 35 cycles at 96°C (1 min), annealing (30 sec) at 66°C for HA or 70°C for NP, and extension at 72°C (30 sec) in a T3-Thermocycler (Biometra, Germany). The amplification of the *LacZ* gene fragment was performed as described [46]. PCR amplicons were detected by electrophoresis using 0.8 % (w/v) agarose-ethidium bromide gels.

### Antibodies

Specific detection of H5 HA was accomplished with the mouse monoclonal antibody (mAb) 15A3 (Rockland, USA) and the polyclonal rabbit LGL antiserum (kindly provided by M. Büttner, Bavarian Health and Food Safety Authority, Oberschleissheim, Germany). The mouse mAb 2442 (Abnova, Germany) was used for specific recognition of the NP protein. The mAb 4D9 [52] allowed detection of the ORFV major envelope protein (F1L), the β-actin specific antibody was purchased from Sigma-Aldrich (Germany). Goat anti-mouse Alexa Fluor 488-conjugated (Fisher Scientific, Invitrogen, Germany), horseradish peroxidase-conjugated anti-rabbit and anti-mouse IgG (Dianova, Germany) and goat anti-mouse fluorescein isothiocyanate (FITC)-conjugated antibody (Dianova, Germany) were used as second antibodies.

### Western blot analysis

Non-infected or infected Vero cells were suspended in 1% (v/v) Triton-X100 (Sigma-Aldrich, Germany) in PBS and incubated for 30 minutes at 4°C. Western blot analysis was performed as described [46]. Protein concentration of the lysates was determined using Pierce BCA Protein Assay kit (Thermo Fisher Scientific, Germany) according to the recommendation of the manufacturer. Afterwards, the lysates were adjusted to equal protein concentrations. The antibodies were diluted in 1X RotiBlock (Roth, Germany) and the substrate Immobilion Western HRP (Millipore, Germany) was used for enhanced chemiluminescence (ECL). X-ray films for ECL were purchased from Pierce (Thermo Fisher Scientific, Germany).

### Immune peroxidase monolayer assay (IPMA)

Expression of inserted HA and NP genes in recombinant-infected cells was demonstrated by IPMA exactly as described [42] using HRP substrate (Vector NovaRED, USA).

### Immunofluorescence

Vero cells infected with the ORFV recombinants were grown in chamber slides (BD Biosciences, Germany), fixed with 2% (v/v) methanol-free formaldehyde (Pierce, Thermo Fisher Scientific, Germany) in PBS and permeabilized with 0.2% Triton-X100 (Sigma, Germany) as reported [46]. Microscopy was performed with ApoTome confocal fluorescence microscope (Axiovert 200 M; Zeiss, Germany) and AxioVision Rel. 4.8 software (Zeiss).

### Flow cytometry

Vero cells were harvested by trypsinization and washed once with FACS buffer (10% v/v foetal bovine serum, 0.1% v/v sodium azide in PBS). Approximately 10^6^ cells were stained with H5 HA-specific primary antibody mAb15A3 for 30 minutes at 4°C. After three times washing the cells were stained in the dark with FITC-conjugated secondary antibody for another 30 minutes at 4°C. To exclude nonviable cells staining with 7-AAD (7-Amino-Actinomycin D; BD Bioscience, Germany) was performed 10 minutes prior to flow cytometry with FACSCalibur (BD Bioscience, Germany) and CellQuest Pro (BD Bioscience, Germany). Gates were set for viable cells negative for 7-AAD.

### Vaccination of mice and Influenza A virus challenge

BALB/c and C57BL/6 mice at the age of 8-12 weeks were obtained from the animal breeding facility of the Friedrich-Loeffler-Institut (Germany). Mice were instilled intranasally (i.n.) under anaesthesia [20] using 50 µl of the indicated mouse 50% lethal dose (MLD50) of HPAIV. For BALB/c mice 1× MLD50 corresponded to 7×10^1^ plaque-forming units (pfu) of strain MB1, 2×10^1^ pfu of strain SN1 and 1×10^4^ pfu of strain PR8. For C57BL/6 mice 2×10^3^ pfu of strain MB1, 1.4×10^3^ pfu of strain PR8 matched to 1× MLD50. Weight loss and survival of infected mice was daily monitored during 14 days after challenge infection. According to the German animal-protection law, animals that lost approximately 25% of their body weight were sacrificed, documented as dead, and thereafter excluded from calculation of the body weight graph. The challenge experiments were performed under BSL3 conditions at the Friedrich-Loeffler-Institut, Tübingen (Germany).

### *In vivo* depletion of T-cell subpopulations

Monoclonal antibodies directed against murine CD4 (mAB YTS 191.1) or CD8 (mAB YTS 169.4) [53] were kindly provided by L. Stitz, (Friedrich-Loeffler-Institut, Germany) and used for depletion of T-cell subsets as described recently [42]. The 1∶25 diluted mAbs were administered intraperitoneally, and 0.2 ml of each mAb was applied per mouse or 0.4 ml of an equal mixture of both mAb for the simultaneous depletion of CD4- and CD8-positive T-cells. The timeline of the injections of mAb is detailed in the Results part. Efficacy of depletion and kinetic of T-cell repopulation was monitored by flow cytometry in a preliminary experiment. Blood was taken from the retro-orbital plexus 2, 6, 9, and 14 days after antibody-treatment at days −2 and 0. The gated lymphocytes were used for double-staining with PE- or FITC-labelled CD3- and CD4- or CD3- and CD8-specific antibodies (BD Biosciences, Germany). Non-depleted mice contained on the average approximately 30 % CD-4 positive T-cells (n = 4: 20.6%–33.5%) and 5 % CD-8 positive T-cells (n = 4: 3.8%–5.9%), respectively. After antibody treatment more than 99% of each T-cell subpopulation remained absent for at least 9 days, before T-cell repopulation started (Table 1), similarly as reported earlier [53], [54].

**Table 1:** Monitoring the success of T-cell depletion.

Days a)	CD4-positive T-cells (%)	CD8-positive T-cells (%)
2	0.20 (n = 4: 0.00–0.80) b)	0.03 (n = 5: 0.00–0.07)
6	0.15 (n = 4: 0.00–0.39)	0.03 (n = 5: 0.00–0.08)
9	0.06 (n = 4: 0.01–0.06)	0.01 (n = 5: 0.00–0.04)
14	1.55 (n = 4: 0.34–3.10)	0.22 (n = 5: 0.06–0.45)

a) Days after second antibody treatment.

b) Mean percentage; number of animals (n) and range of percentage is given in parenthesis.

The mice (BALB/c; n = 8) were i.m. immunized twice (14 days interval) with 10^7^ pfu of the recombinant D1701-V-HAh5n. Fourteen days after last vaccination the i.n. challenge infection was performed with 20× MLD_50_ HPAIV MB1.

### Hemagglutination inhibition (HI) test

HI test was performed according to the OIE (World Organization for Animal health) instruction manual (Manual of diagnostic tests and Vaccines for terrestrial animals, 5^th^ edition, 2004) in 96-well microtiter plates (Greiner bio-one, Germany) using 25 µl 1% (v/v) suspension of chicken red blood cells in PBS. Twenty-five μl of two-fold dilutions of heat-inactivated (30 min, 56°C) sera were incubated for 40 min at RT with 4 hemagglutination units (HAU) of formalin-inactivated H5N1 MB1 virus. The HI titre was defined as the reciprocal of the highest serum dilution inhibiting hemagglutination.

## Results

### Generation of the new ORFV recombinants

**Figure 1:**
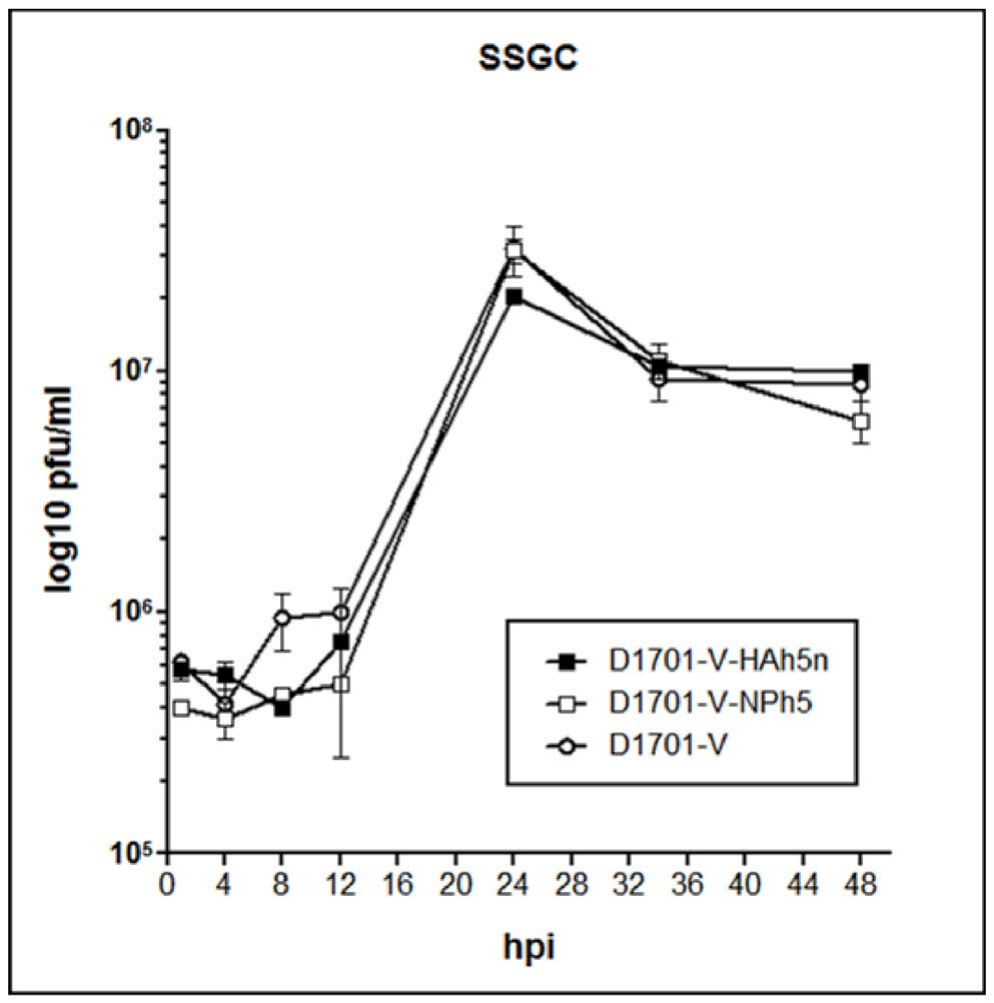
Single step growth curve. Comparison of the *in vitro* growth characteristics of D1701-V-HAh5n, D1701-V-NPh5 and parental D1701-V. Vero cells were infected with moi 5.0 and total cell lysates were taken for virus titration at the indicated hours post infection (hpi). The results demonstrate very similar growth kinetics of both ORFV recombinants and the parental D1701-V.

**Figure 2:**
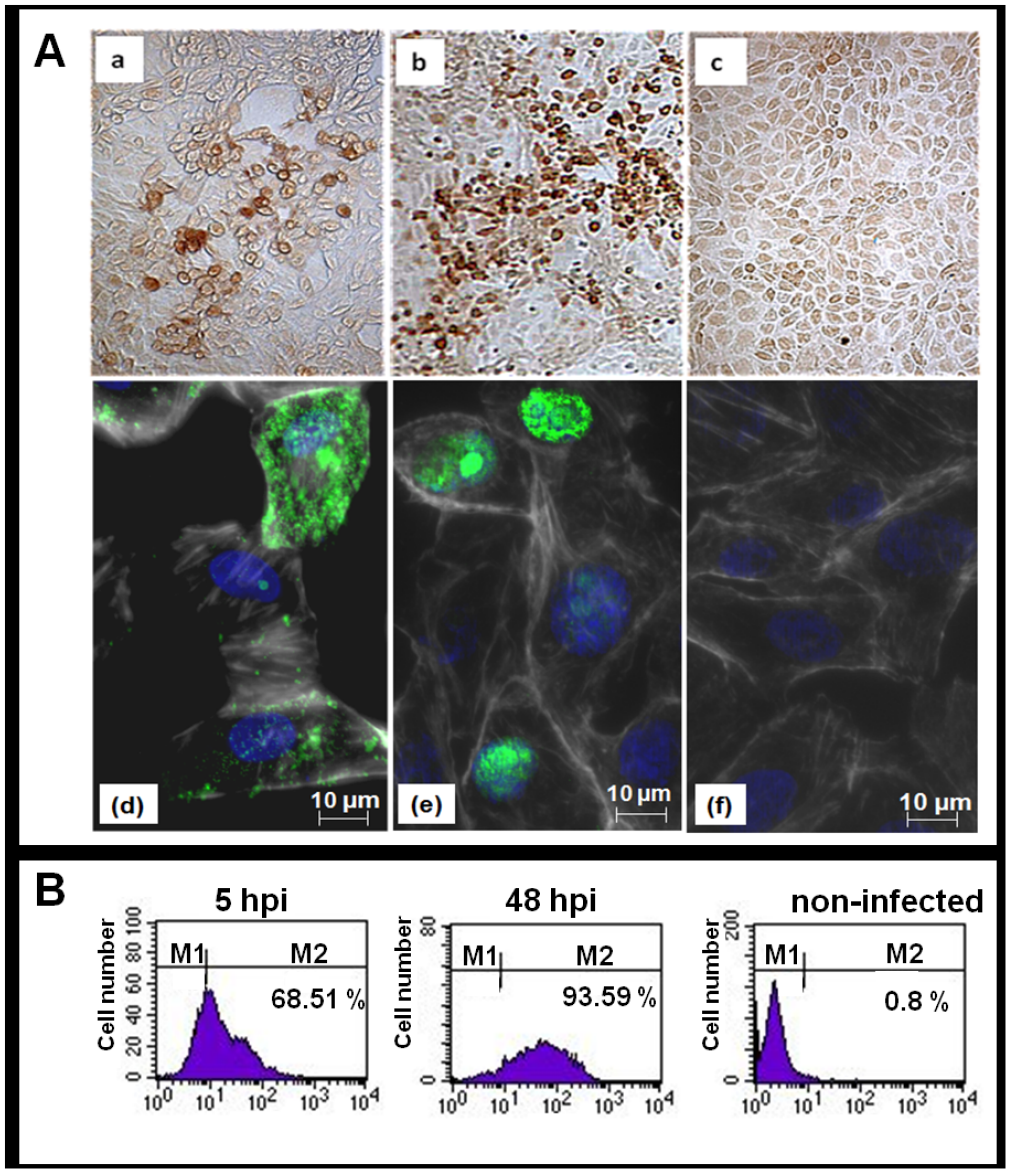
AIV gene expression of D1701-V-HAh5n and D1701-V-NPh5. **(A)** Expression of HA and NP in ORFV recombinant-infected cells demonstrated by IPMA (panel a–c) and by immunofluorescence (panel d–f). Vero cells were infected with D1701-V-HAh5n (panel a and d), D1701-V-NPh5 (panel b and e) or non-infected (panel c and f). Three days post infection transgene expression (brown) is detected with the HA-specific, 1∶250 diluted LGL antiserum (panel a, magnification ×40) and with the NP-specific, 1∶ 500 diluted mAb 2442 (panel b, magnification ×20), whereas non-infected cells (panel c, magnification ×40) remained unstained. HA-specific immunofluorescence (green) is shown 24 hpi with the 1∶250 diluted mAb 15A3 (panel d), and nuclear NP expression with the 1∶1,000 diluted mAb 2442 (panel e). Non-infected cells as negative control are depicted in panel f. The cell nuclei are DAPI-stained (blue) and the actin cell skeleton is stained by Phalloidin-CF647 (white). **(B)** Cell surface expression of H5 HA was quantified by flow cytometry. Vero cells were harvested 5 hours (5 hpi) and 48 hours (48 hpi) after D1701-V-HAh5n infection (moi 1.0) and stained with mAb 15A3. The histograms show the cell number (ordinate) plotted against the fluorescence intensity (abscissa) gated for 7-AAD negative, viable cells. HA-positive cells are gated in M2, negative cells in M1. Distinct H5 HA cell surface expression was demonstrable already 5 hpi increasing with later times after infection.

The transfer plasmids containing the H5 HA gene (pdV-HAh5n3) or the NP gene (pdV-NPh5n) were used for electroporation of Vero cells infected with D1701-VrV, which expresses the *LacZ* gene and enables the blue-white selection as described [44], [46]. New white ORFV recombinants were selected by PCR as described in Material and Methods. Single plaque virus isolates of D1701-V-HAh5n and D1701-V-NPh5 were subject to five and four additional rounds of plaque purification, respectively, to obtain genetically homogeneous new ORFV recombinants. Single step growth curve experiments demonstrated that the insertion of the H5 HA or NP gene had no influence on the *in vitro* growth characteristics of both ORFV recombinants compared to the parental ORFV D1701-V (Fig. 1). Transgene expression of the virus plaque isolates was tested by IPMA. As shown in Figure 2A (panel a–c) expression of the H5 HA and NP transgene in ORFV recombinant-infected cells was demonstrable by specific brown immune staining (Fig. 2A, panel a, b), but not in non-infected cells (Fig. 2A, panel c) or in cells infected with the parental D1701-VrV (data not shown). Correct insertion of the HA or NP gene into the *vegf-e* gene locus of D1701-V was verified by PCR and Southern blot hybridization of recombinant virus DNA (data not shown).

### Expression of the H5 HA and NP gene

Indirect immunofluorescence assays demonstrated expression of H5 HA and NP gene in recombinant virus-infected cells (Fig. 2A). As expected, the NP gene was expressed in the nuclei of D1701-V-NPh5n-infected cells (Fig. 2A, panel e), whereas non-infected (Fig. 2A, panel f) or parental virus-infected Vero cells (data not shown) remained negative. Cell surface expression of H5 HA was further demonstrated by flow cytometry of D1701-V-HAh5n-infected Vero cells. Already five hours after infection (hpi) 68.5% of the infected cells expressed the H5 HA on the cell surface increasing to 93.6% at 48 hpi (Fig. 2B).

**Figure 3:**
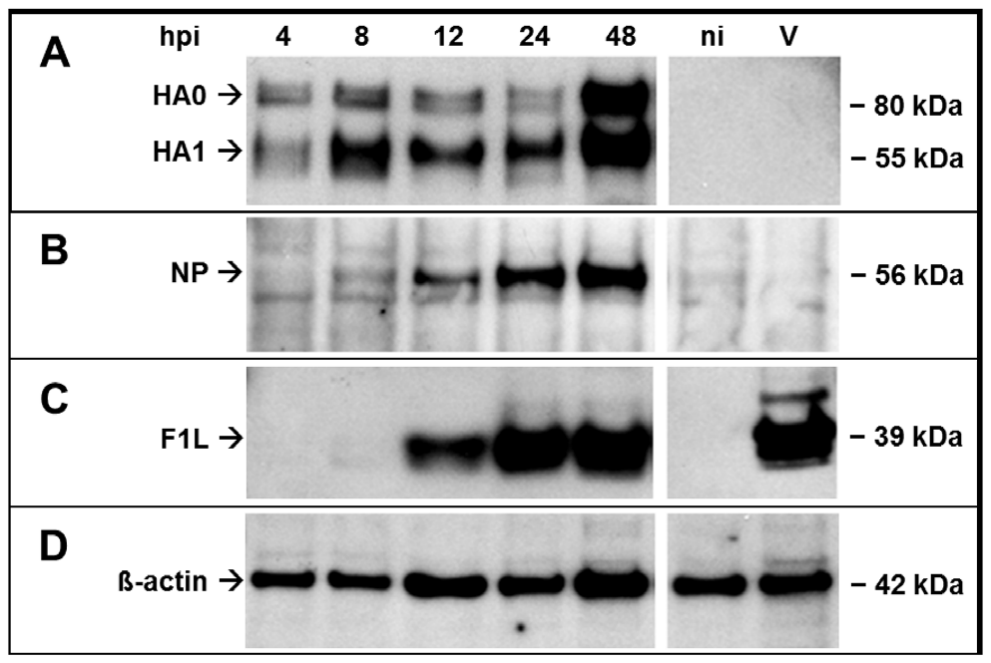
Western Blot analysis for the detection of HA and NP. Cell lysates were harvested at indicated hours post infection (hpi) with **(A, C)** D1701-V-HAh5n (moi 3.0) or **(B, D)** D1701-V-NPh5 (moi 1.0). As controls non-infected cells (lanes ni) or parental D1701-V- (moi 1.0) infected cells (lanes V) were tested at 24 hpi. **(A)** H5 HA was detected with the specific mAb 15A3 (diluted 1∶5,000), **(B)** NP was detected with specific mAb 2442 (diluted 1∶2,000). **(C)** The mAb 4D9 (diluted 1∶800) was used to detect the ORFV major envelope protein F1L expressed at late times pi. **(D)** Beta-actin was demonstrated as loading control. The apparent mol. wt. of the specific proteins is indicated in kilodalton (kDa).

Expression of the inserted influenza A virus HA and NP gene was also inspected by Western blot analysis at different times after infection of Vero cells with D1701-V-HAh5n or D1701-V-NPh5. Using mAb 15A3 the expression of the H5 HA was detectable from 4 hpi onwards with increasing amounts at later times after infection (Fig. 3A). At all tested time points after infection the precursor protein HA0 was recognized as a double band migrating with a mol. wt. of approximately 80 kDa as well as the subunit HA1 (55 kDa). The subunit HA2 is not recognized by the used antibody. Non-infected (ni) cells and cells infected with parental D1701-V (V) remained negative. The NP protein (56 kDa) was demonstrable with mAb 2242 by Western blot analysis (Fig. 3B). In the presented experiment, protein lysates were obtained from cells infected with a lower moi (1.0) of D1701-V-NPh5 in contrast to the D1701-V-HAh5n lysates (moi = 3.0), which explains the weaker NP expression compared to HA. In addition, early NP expression could be unequivocally proven by Northern blot analysis (data not shown). The late ORFV major envelope protein F1L (39 kDa) was recognized beyond 12 hpi with the mAb 4D9, which reflected multiplication of the ORFV recombinants in Vero cells (Fig. 3C). Nearly comparable protein loading was verified by detection of cellular β-actin (Fig. 3D).

### Protection of mice from challenge with divergent H5N1 strains

**Figure 4:**
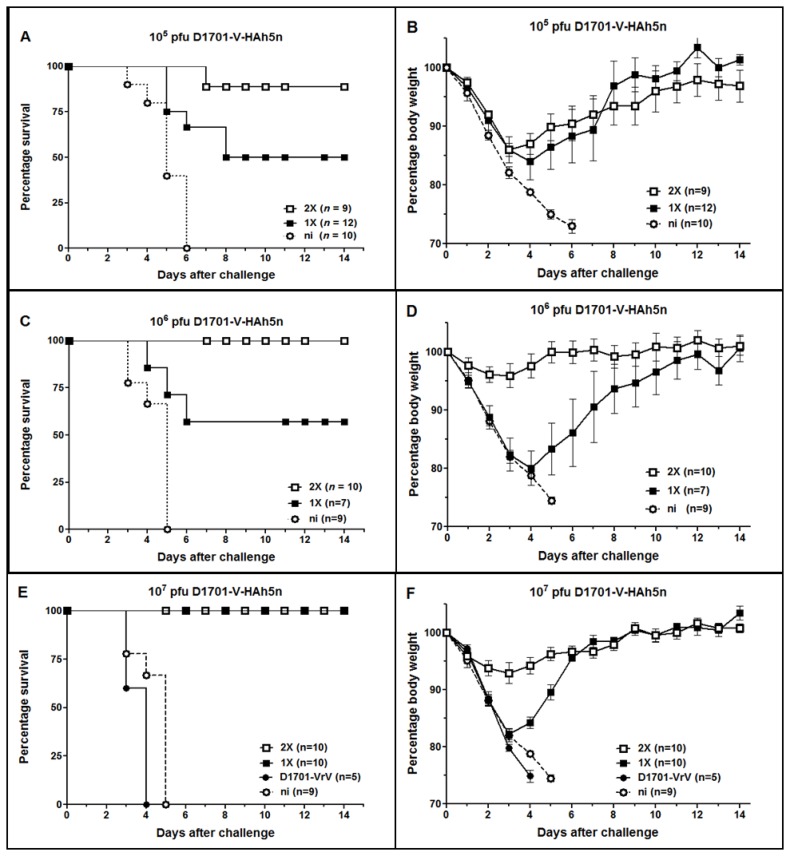
Protective efficacy of D1701-V-HAh5n in C57BL/6 mice. Survival rates (A, C, E) and mean body weight changes (B, D, F) of C57BL/6 mice i.m. immunized once (1X) or twice (2X) with the indicated pfu of D1701-V-HAh5n. The mice were monitored during 14 days after i.n. challenge infection with 20× MLD50 of H5N1 strain MB1. As controls mice were immunized two times with 10^7^ pfu of parental ORFV D1701-VrV (E, F) or were non-immunized (ni). SEM is shown by bars, *n* indicates the total number of mice in each group. Mice exhibiting more than 25% loss of body weight were sacrificed according to the German animal protection law.

The protective potential of the new ORFV recombinants was evaluated first in C57BL/6 mice after i.m. immunization with different doses followed by i.n. challenge infection with strain MB1 (20× MLD50). A single immunization with 1×10^5^ pfu of D1701-V-HAh5n was not able to mediate proper protection against the MB1 challenge infection. Except of 2 mice, all animals suffered from severe disease and 6 out of the 12 mice had to be euthanized at days 6 to 8 after challenge (Fig. 4A). The surviving mice gradually lost body weight 2 to 4 days after challenge (Fig. 4B), individually ranging from 8% to 15%, and one animal lost 24% of weight. Thereafter all survivors recovered and regained their body weight (Fig. 4B). A booster immunization with 10^5^ pfu improved protection rate to 89% survival, 8 out of 9 mice survived the challenge (Fig. 4A). One survivor suffered from severe illness associated with 24% weight loss before recovering, whereas the body weight loss of the other 7 mice ranged only from 8% to 17% around 3 days after challenge (Fig. 4B, mean 12%). Increasing the immunization dosage of D1701-V-HAh5n to 1×10^6^ pfu, again as a single application mediated only partial protection from challenge (Fig. 4C) and from body weight loss (Fig. 4D). The 4 surviving animals experienced weight loss of 7%, 11%, 21%, and 24%, respectively, before recovering beyond day 4 (Fig. 4D). Two immunizations with 1×10^6^ pfu, however, conferred complete protection from lethal challenge (Fig. 4C and 4D). Only 3 mice lost 11% body weight, whereas the other mice sustained their body weight and remained healthy.

The administration of 10^7^ pfu of D1701-V-HAh5n clearly improved the generation of protective immunity against 20× MLD50 MB1 challenge. Single vaccination with that immunization dosage was sufficient to mediate 100% survival (Fig. 4E). At day 3 after challenge a mean body weight loss of 18% was observed (individually ranging from 14% to 23%), but thereafter all mice fully recovered and regained their body weight (Fig. 4F). All C57BL/6 mice receiving 2 doses of 1×10^7^ pfu of that recombinant survived the challenge (Fig. 4E) and stayed healthy (Fig. 4F). Only 2 animals exhibited a transient weight loss of 17% until day 3 before also retrieving their original body weight. Control mice receiving 2 injections of 10^7^ pfu of the parental ORFV D1701-VrV were not protected against the MB1 challenge infection. Onset of disease and loss of body weight were similar to non-immunized animals, and the mice had to be euthanized 4 days after challenge (Fig. 4E and F).

The NP gene expressing recombinant D1701-V-NPh5 did not confer protection from lethal challenge or prevent morbidity after infection with 20× MLD50 MB1. Groups of mice (C57BL/6, n = 10) were i.m. immunized once or twice and neither 10^6^ nor 10^7^ pfu of D1701-V-NPh5n were able to protect. All animals became diseased and had to be sacrificed during days 4 to 6 after challenge, likewise the non-immunized or parental D1701-VrV vaccinated control animals. Also three doses of 5×10^6^ pfu did not confer protection against challenge infection (data not shown).

**Figure 5:**
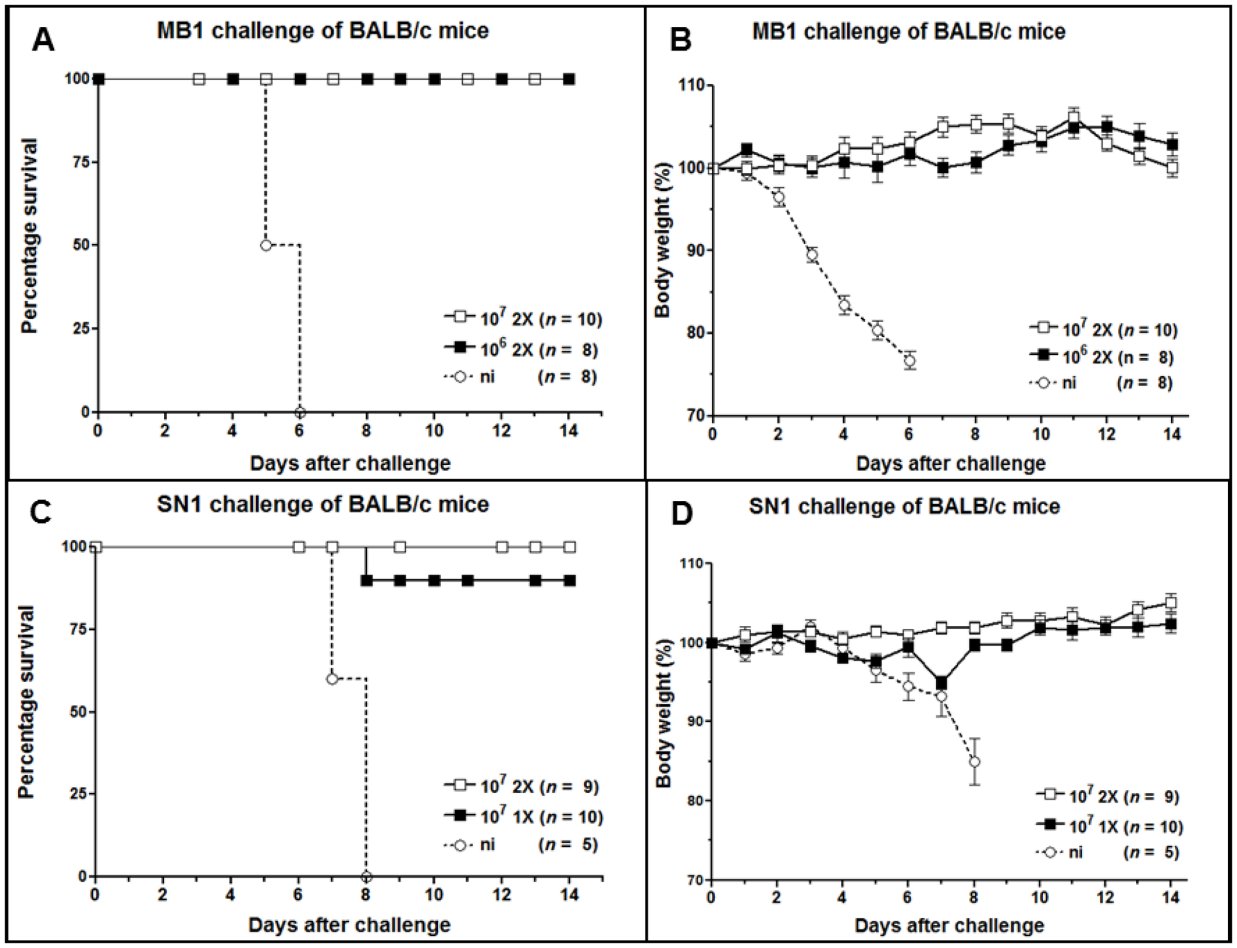
Protection of BALB/c mice from cross-clade HPAIV challenge infection. Survival rates (A, C) and body weight changes (B, D) of BALB/c mice after i.n. challenge with 20× MLD50 of H5N1 strain MB1 (A, B) or SN1 (C, D). Single (1X) or booster (2X) i.m. immunization was performed with 10^6^ pfu or 10^7^ pfu D1701-V-HAh5n and mice were monitored during 14 days after challenge infection. Mean percentage of body weight change is shown (bars indicate SEM), and *n* indicates the number of mice per group. After approximately 25% loss of body weight mice were sacrificed according to the German animal protection law.

The protective potential of D1701-V-HAh5n was also tested in BALB/c mice, which are approximately 30-fold more susceptible to MB1 challenge as compared to C57BL/6 mice (see Material and Methods). Two i.m. applications of either 1×10^6^ or 1×10^7^ pfu also elicited complete protection against 20× MLD50 of strain MB1. All immunized animals survived the lethal challenge without body weight loss or any sign of disease, in contrast to all non-immunized mice (Fig. 5A and B). As for C57BL/6 mice again vaccination with D1701-V-HAh5n, expressing H5 HA from a clade 1 HPAIV (A/Vietnam/1203/2004), could protect from a cross-clade challenge infection with HPAIV strain MB1 (clade 2.2.1). Finally we found protection of mice from challenge with 20× MLD50 HPAIV strain SN1, which belongs to influenza A virus clade 2.2.3. As depicted in Figure 5C, 9 out of 10 BALB/c mice survived the challenge infection after a single administration of 10^7^ pfu D1701-V-HAh5n, and all animals immunized twice survived the challenge. Both groups of mice did not show loss of body weight (Fig. 5D) and the non-immunized mice had to be sacrificed within 8 days after challenge infection.

### Protection from challenge with H1N1 strain PR8

**Figure 6:**
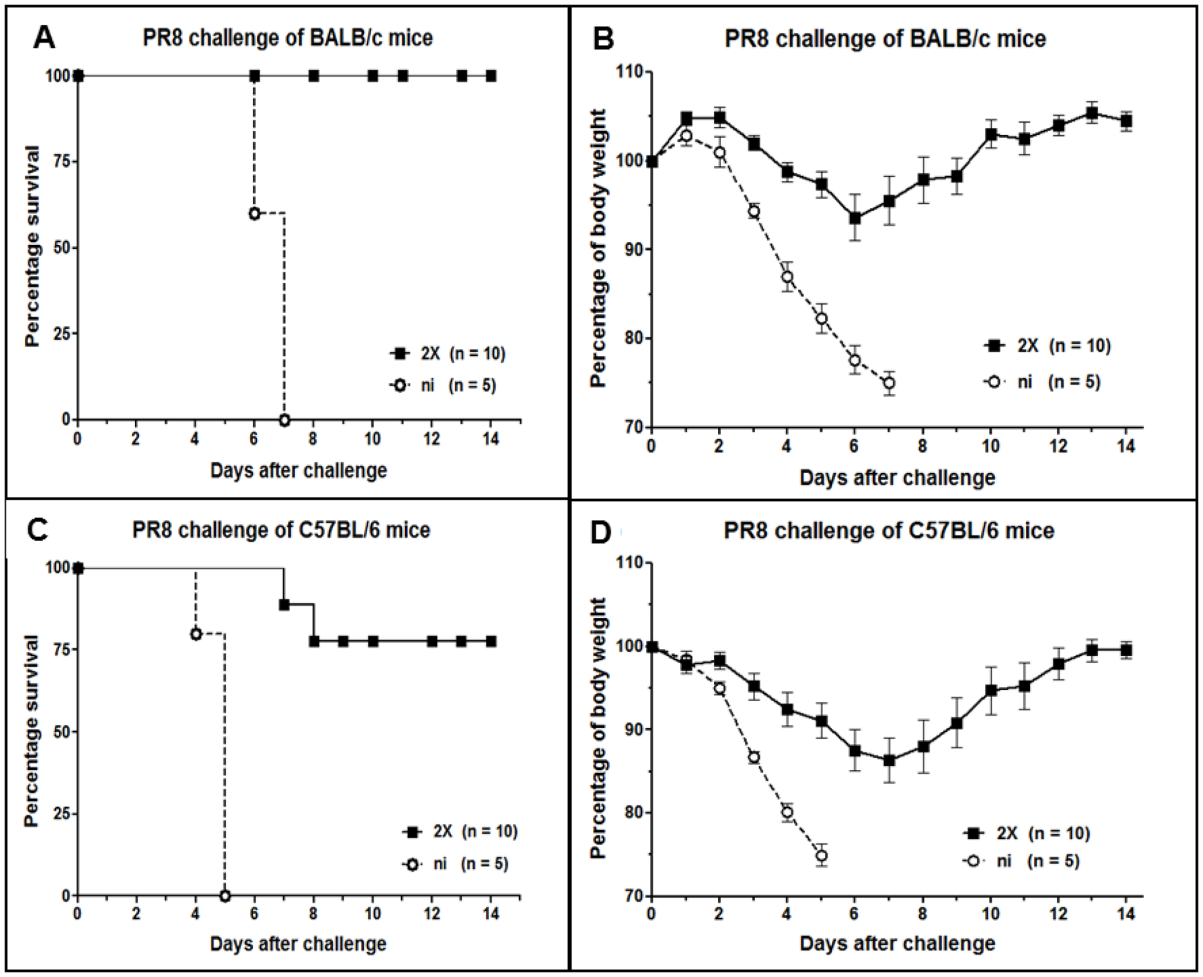
Protection against heterologous influenza A virus H1N1. Survival rates (A, C) and average body weight loss (B, D) of BALB/c (A, B) or C57BL/6 (C, D) mice i.m. immunized twice (2X) with 10^7^ pfu D1701-V-HAh5n. The percentage of body weight (bars indicate SEM) was monitored during 14 days after i.n. challenge infection with 50× MLD50 (A, B) or 20× MLD50 (C, D) of H1N1 strain PR8. Mice suffering from more than 25% body weight loss were sacrificed. Control non-immunized mice (ni) are shown, *n* indicates the number of mice per group.

After demonstrating the induction of protective immunity against HPAIV H5N1 strains of clade 2, we tested the potential of the recombinant D1701-V-HAh5n to protect mice against heterologous H1N1 influenza A virus. BALB/c and C57BL/6 mice were i.m. immunized once or twice with 10^7^ pfu of D1701-V-HAh5n followed by i.n. challenge infection with lethal doses of the H1N1 strain PR8. Whereas a single immunization was not sufficient to protect BALB/c mice (data not shown), after two vaccinations all BALB/c mice survived the challenge using 50× MLD50 (Fig. 6A). Transient, slight loss of body weight ranging from 5% to 17% was observed with 6 out of the 10 mice followed by complete recovery during days 7 to 10 (Fig. 6B). On the contrary, 2 out of the 10 double-immunized C57BL/6 mice were not protected from the 20× MLD50 PR8 challenge (Fig. 6C). Six out of the 8 survivors exhibited weight losses at days 6 to 9 (individually ranging from 8% to 22%) before completely recovering (Fig. 6D). In contrast to H5N1 strain MB1 (Fig. 4), the peak of disease was delayed by 4–5 days after challenge infection with H1N1 strain PR8 (Fig. 6). The most pronounced body weight loss was found around day 6–7 (BALB/c mice) or day 7–8 (C57BL/6 mice) after PR8 challenge, but at day 3 after MB1 challenge.

Taken together, two immunizations with the H5 HA-expressing ORFV recombinant mediated potent protection of BALB/c or C57BL/6 mice against cross-clade strains MB1 or SN1, and against the human H1N1 strain PR8.

### HA-specific serum antibody response

**Figure 7:**
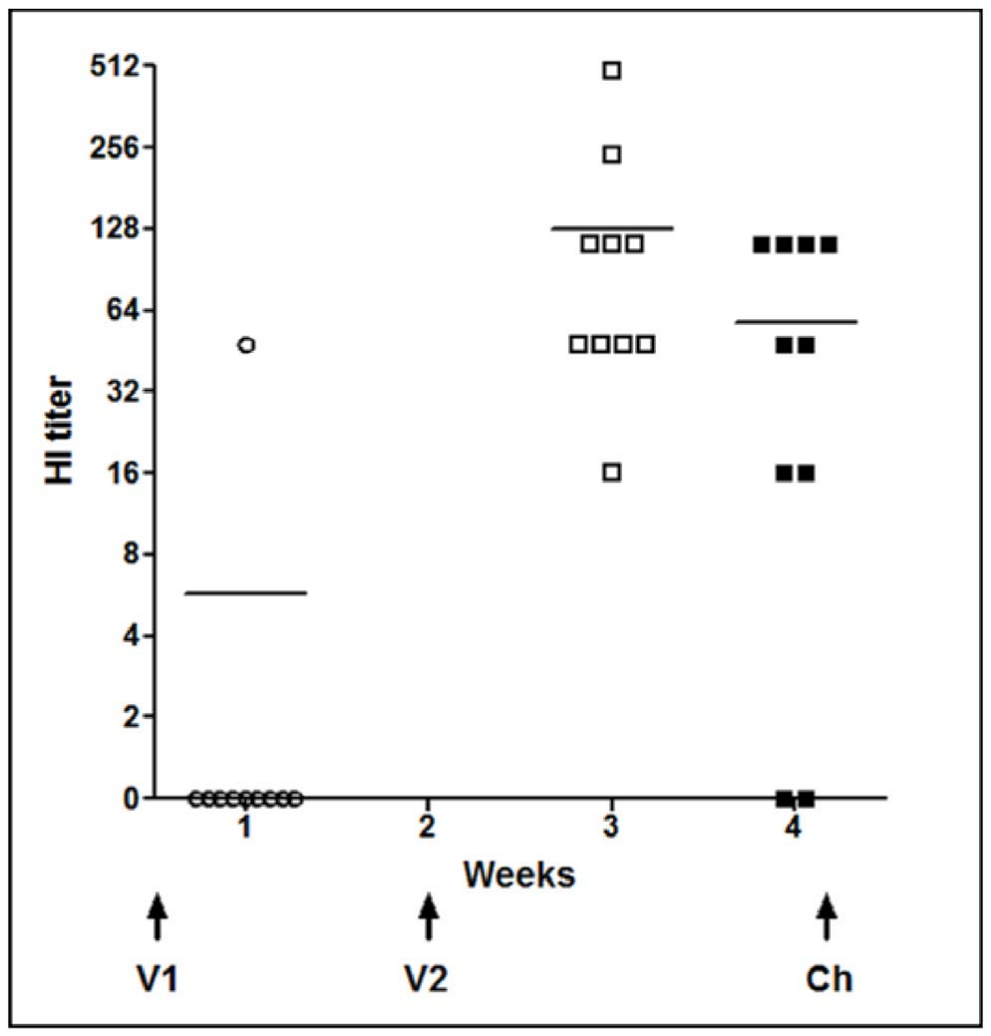
HA-specific serum antibody response. H5 HA-specific serum antibody response of mice elicited after i.m. immunization with 10^7^ pfu of D1701-V-HAh5n. The hemagglutination inhibition (HI) titers (reciprocal log2) of individual mice were determined 1 week after prime immunization (V1), after booster immunization (V2), and at the day of challenge infection (Ch). Sera from control mice immunized twice with 10^7^ pfu of D1701-VrV (VrV) displayed unspecific HI titers of 1∶16. The lines denote the mean titers calculated from the individual sera.

The immune response stimulated by D1701-V-HAh5n immunization of C57BL/6 mice was determined by HI tests as described in *Material and Methods*. Figure 7 exemplary demonstrates the HI titres obtained by the i.m. application of 10^7^ pfu of D1701-V-HAh5n. One week after prime immunization relevant HA-specific antibodies were detectable in the serum of only one out of 10 mice, but one week after booster immunization all animals except of one had seroconverted (Fig. 7, week 3). The titres of the individual sera ranged between 1∶32 and 1∶512 resulting in a mean titre of 1∶144. Thereafter, only a slight decline to a mean titre of 1∶74 was found just before challenge infection (Fig. 7, Ch). Very similar HI titres were also found after applying 10^6^ pfu of D1701-V-HAh5n (data not shown), whereas after immunization with 10^5^ pfu no specific immune response could be detected. Sera from non-immunized mice (data not shown) or control mice immunized twice with 10^7^ pfu of the parental virus D1701-VrV exhibited unspecific HI titres of 1∶16 (Fig. 7, VrV). In summary, the stimulation of a distinct cross-clade HA serum antibody response needed two immunizations with 10^6^ or 10^7^ pfu of D1701-V-HAh5n.

### Importance of T-cell subsets in D1701-V-HAh5n-immunized mice

**Figure 8:**
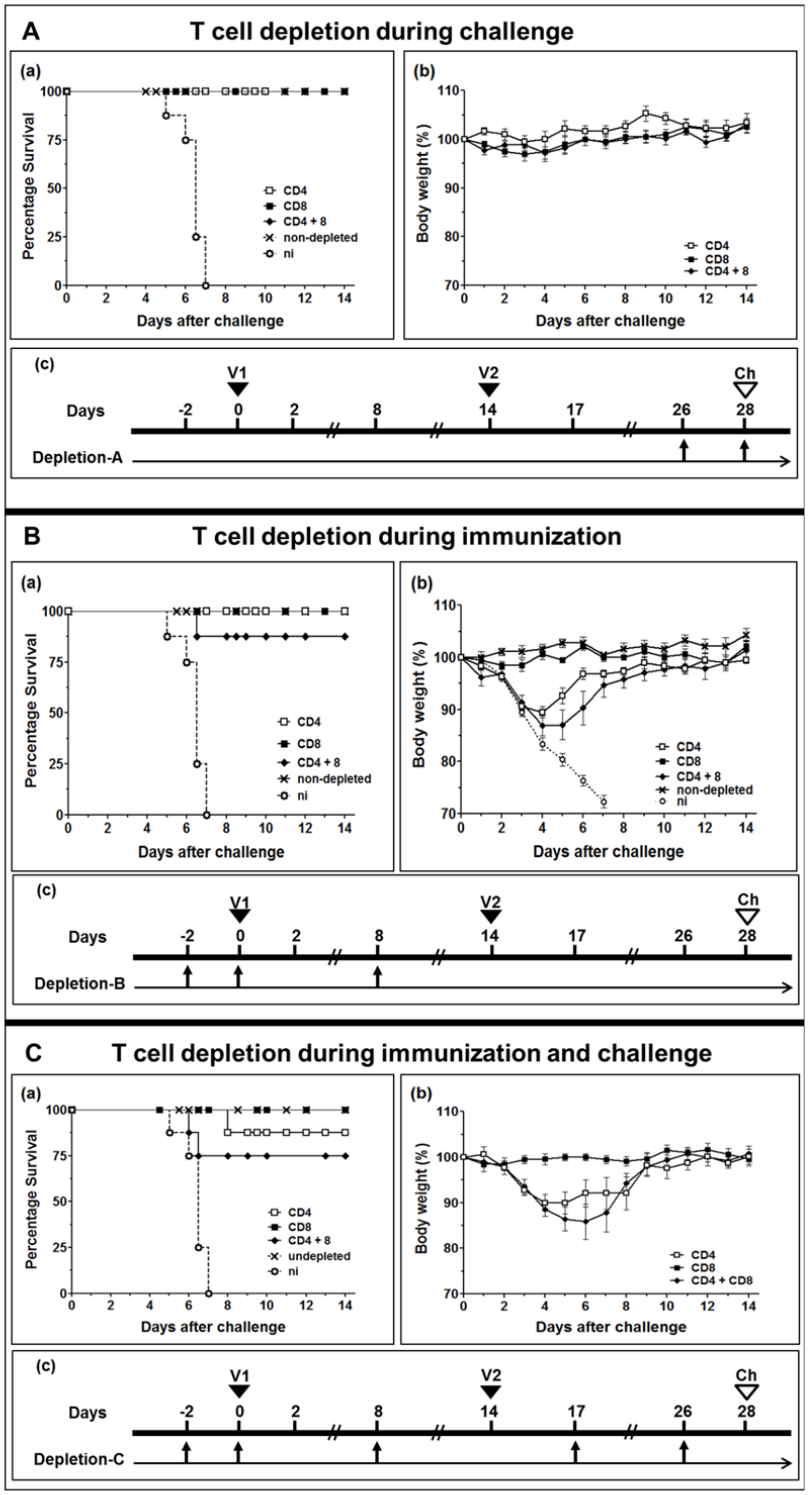
Role of T-cells for D1701-V-HAh5n-induced protection. BALB/c mice were immunized twice (V1 and V2) with 10^7^ pfu of D1701-V-HAh5n and depletion was performed during immunization **(A)**, during challenge **(B)** or during immunization and challenge **(C)**. Challenge (Ch) was performed with 20× MLD50 strain MB1 and mice were monitored during 14 days after challenge. Survival of mice (*n* = 8) is shown in panels a, the mean percentage of body weight (bars indicate SEM) is demonstrated in panels b. Mice were sacrificed after having dropped more than 25% of their original body weight. Panels c schematically depict the days of *in vivo* depletion of CD4-positive, CD8-positive or both CD4- and CD8-positive T-cell subset as described in *Material and Methods*. For control, challenge infection of immunized non-depleted animals or non-immunized mice (ni) was also performed.

To examine the relevance of T-cells for the D1701-V-HAh5n mediated immunity the CD4-positive and/or CD8-positive T-cell subsets of BALB/c mice were depleted *in vivo*. Flow cytometry ensured successful depletion of the T-cells (data not shown). The T-cell populations were removed as described in *Materials and Methods* and depicted in Figure 8, panels c. At first we examined the relevance of T-cells present during H5N1 challenge infection (Fig. 8A, Depletion-A, panel c). In this situation the lack of CD4- and/or of CD8-positive T-cells had no influence on the protective immunity generated by D1701-V-HAh5n. All animals of the 3 groups survived without body weight loss (Fig. 8A, panel a and b).

Next, we analysed the importance of the T-cell subsets for priming the protective anti-HA response (Fig. 8B, depletion-B). After eliminating CD4- or CD8-positive T-cells all immunized mice survived the challenge infection, and after simultaneous removal of both T-cell subsets still 7 out of 8 mice resisted the challenge (Fig. 8B, panel a). All challenged animals depleted for CD8-positive T-cells during immunization retained their body weight (Fig. 8B, panel b). The lack of CD4-positive T-cells during prime immunization resulted in slight decrease of body weight, ranging from 8% to 18%, during days 3–5 after challenge (Fig. 8B, panel b). Thereafter all animals recovered and regained their original body weight (Fig. 8B, panel b). A similar effect was observed after removing both CD4- and CD8-positive T-cells. Five out of the 7 surviving mice showed weight losses ranging from 9% to 21% around days 4–5 after challenge (Fig. 8B, panel b).

Finally, we tested the effect of the absence of CD4- and/or CD8-positive cells during immunization and challenge infection (Fig. 8C, Depletion-C, panel c). Again, all mice missing only CD8-positive T-cells survived the lethal challenge without loss of body weight (Fig. 8C, panel a, b). After depletion of CD4-positive cells 7 out of 8 immunized mice survived the challenge, one animal exhibited 23% weight loss, the three other mice lost weight between 11% and 14% at days 4 and 5 after challenge (Fig. 8C). Two out of 8 mice did not survive challenge infection after combined removal of CD4- and CD8-positive T-cells (Fig. 8C, panel a). Four mice lost 17% to 22% of body weight at day 6 after challenge before completely recovering (Fig. 8C, panel b). Collectively, the presented results implicate the importance of CD4-positive T-cells for eliciting a robust, protective immunity in mice by the use of the new H5 HA-expressing ORFV recombinant.

## Discussion

In previous studies we demonstrated the utility of recombinant ORFV vectored vaccines [42], [43], [44], [45], [46]. Here we describe the generation and evaluation of two new ORFV recombinants, which express the H5 HA gene or the NP gene of H5N1 HPAIV. Both AIV genes were expressed under the control of the early promoter of the ORFV vegf-e gene, which allows expression of the inserted genes without the need of recombinant virus multiplication as also reported for other ORFV recombinants [42], [44]. In the ORFV-permissive Vero cell line both recombinants demonstrate comparable virus growth kinetics to each other and also to the parental virus D1701-V used for recombinant virus generation. Western blot and immunofluorescence analyses demonstrated correct expression of both genes including the cleavage of the HA precursor protein HA0. The H5 HA was also demonstrable on the surface of the ORFV recombinant infected cells, and the NP protein was expressed in the cell nucleus. HA and NP genes were chosen because both are of importance for the induction of a protective immune response. The crucial role of HA-specific virus-neutralizing antibodies for protection has been manifold documented. We used the H5 HA from influenza strain A/Vietnam/1203/ 2004, because most H5N1 vaccines based on the HA from the highly virulent human isolates of this Vietnam strain confer solid protection against H5N1 strains [38]. The contribution of the conserved NP antigen to protection is mainly attributed to the activation of cellular immune responses including the induction of specific cytolytic T-cells [23], [55], [56]. Additionally, it was reported that NP-specific antibodies can exert potent antiviral activity [55].

The protective capacity of the new ORFV recombinants was assessed in the mouse challenge model. The results showed that the ORFV recombinant expressing the conserved NP was not protective in mice. None of the animals survived the lethal challenge infection, also not after three immunizations with the recombinant. It remains to be determined whether the lack of protection can be explained by insufficient activation of T-cells or dendritic cells and/or missing induction of specific antibodies. Both B- and T-cells were reported to be of importance for NP-mediated protection [55]. Another explanation might be that the immunity mediated by D1701-V-NPh5 was not sufficient to protect from the dose of lethal challenge virus used in our experiments. Reduced protective efficacy of NP against increasing challenge virus dose was found in mice and ferrets [57]. Although non-protective the described experiments do not exclude the utility of D1701-V-NPh5 in prime-boost vaccination regimens, similarly as reported for DNA prime-recombinant adenoviral boost immunization with NP [58].

The H5 HA-expressing recombinant D1701-V-HAh5n was found to elicit very good protection in a dose-dependent manner. The results showed that booster i.m. immunization was superior to single application of the recombinant. The animals of both mouse strains used were protected not only from lethal challenge but also from disease according to weight loss determination. All mice immunized twice with 10^6^ or 10^7^ pfu of the recombinant survived and remained healthy after challenge infection with two different cross-clade H5N1 strains, namely MB1 (clade 2.2.1) and SN1 (clade 2.2.3). Similar findings were reported for MVA recombinants expressing the H5 HA of clade 1, which were able to protect mice against infection with H5N1 AIV of clades 2.1.3, 2.2 and 2.3.4 [39]. Despite only 66% HA amino acid homology, the presented recombinant D1701-V-HAh5n was able to protect BALB/c and C57BL/6 mice against the heterologous AIV strain PR8 (H1N1). The results indicate slightly better protection of BALB/c mice, which resisted a higher challenge virus dose (50× MLD50) compared to C57BL/6 mice (20× MLD50). Whether the different genetic background of the two mouse strains might influence the anti-AIV immune response is not known. The major determinants of the D1701-V-HAh5n induced cross-protective immunity, cross-neutralizing HA antibodies and/or specific cytotoxic T-cells, must be clarified. Both are suspected to work in protection from heterologous AIV infection [59], as also cooperation of virus-specific CD8-positive T-cells and non-neutralizing antibodies was described [60].

The presence of HI antibodies with titres of 1∶40 or higher are considered to predict protection (for review [27]), but animals with low or without detectable HI antibodies were also protected, as for instance by poxvirus based vaccination [61]. The presented data indicate that only the twofold application of higher doses of D1701-V-HAh5n elicited moderate HI antibody responses and protected all animals from disease and death. Similarly it was reported that 2 injections of HA-based vaccines can be necessary to elicit higher HI antibody titres (for review [62]). Also higher doses of MVA-based H5 HA recombinant have been necessary to induce detectable antibodies [41]. Two immunizations with 10^5^ pfu of D1701-V-HAh5n did not induce detectable HI antibodies also suggesting that the magnitude of the antibody responses can depend on the vaccine dose. But nevertheless more than 80% of these animals survived the lethal challenge infection, which implies a certain immune control. In addition, the still not completely unravelled immunomodulating properties of ORFV strain D1701 [63] can be considered to improve not only cross-protective immunity. Collectively, these results might indicate the additional involvement of T-cells for the formation of a protective immunity.

The mouse immunization experiments demonstrated that two injections of D1701-V-HAh5 were beneficial to mediate robust protective immunity from lethal AIV challenge. To investigate the involvement of T-cells in protection, immune mice were *in vivo* depleted of CD4-positive and/or CD8-positive T-cells as described. Elimination of the T-cell subsets after the two vaccinations before challenge infection did not affect protective immunity. That can be explained by the development of a complete robust protective immunity before depleting the T-cell subsets. Most probably specific antibodies present at the time of challenge infection control the virus. The body weight loss indicated that the presence of CD4-positive T-cells at prime vaccination contributed to disease control, although all challenge infected animals recovered (Fig. 8B). The lack of B-cell help by CD4-positive T-cells during prime immunization can be expected to impair the production of antibodies that effectively neutralize the virus [21]. Depletion of CD4-positive T-cells alone or in combination with CD8-positive T-cells during immunization and challenge resulted in loss of body weight and slightly reduced survival rate (Fig. 8C). Most probably the missing CD4-T-cell help until the time of challenge impeded maturation of B-cells and consequently an effective specific antibody response necessary for early control of challenge virus (for review [64]). That can be also suggested from a prolonged course of disease for 2 days. The question on a possible contribution of CD8-positive T-cells for protective immunity mediated by the HA-expressing ORFV recombinant could be answered by the use of B-cell knock-out mice for *in vivo* deletion of T-cell subsets. Whether CD8-positive T-cells add some effector functions and/or cytokine production remains to be investigated in more detail. Conclusively, the presented findings show that CD4-positive T-cells are needed to prime protective immunity, but deleting these T-cell subset later, e.g. before challenge infection, does not substantially reduce protection. This supports recent reports on the importance of CD4-positive T-cells and of specific antibodies for protection from H5N1 and on the minor protective role of CD8-positive T-cells [20]. CD4-positive T-cells are also important for the development of memory B- and T-cells and thus, additionally aid to increase the protective immune response against AIV [22], [28]. Moreover, they help to clear infected cells early after infection also in the absence of CD8-positive T-cells by antibody-independent, cytotoxic mechanisms or interferon-gamma secretion [65] (for review [64]).

In conclusion, the presented study adds another example of the utility of the *Parapoxvirus* ORFV strain D1701-V as a versatile vector virus platform for the development of live non-adjuvanted recombinant vaccines, which can be used for repeated immunizations. The ORFV based vaccines can be easily propagated in the non-tumorigenic, permanent Vero cell line, also accepted for influenza virus vaccine production [66]. The application of the H5 HA-expressing ORFV recombinant to protect against cross-clade HPAIV or heterologous AIV can be of great interest for vaccination of pets that have the potential to transmit H5N1 from domestic animals to humans. HPAIV H5N1 or H7N7 strains have the capacity to cross the species barrier by infecting dogs and domestic cats, respectively (for review [67]), [68], [69]. The excellent applicability and safety of ORFV-vectored vaccines in pets was demonstrated recently [42]. Due to the very good experience of using ORFV recombinants for immunization of pigs [43], [48], [49] the application of AIV gene expressing ORFV recombinants could also represent alternative vaccines for this AIV relevant host. Based on the presented findings more detailed studies must now scrutinize the induced immune response. In addition, improved cross-protective immunity against AIV can be attempted by using additional recombinants expressing other immune-relevant proteins of AIV.
